# Elongation factor P controls translation of the *mgtA* gene encoding a Mg^2+^ transporter during *Salmonella* infection

**DOI:** 10.1002/mbo3.680

**Published:** 2018-06-27

**Authors:** Eunna Choi, Daesil Nam, Jeongjoon Choi, Shinae Park, Jung‐Shin Lee, Eun‐Jin Lee

**Affiliations:** ^1^ Department of Genetic Engineering and Graduate School of Biotechnology College of Life Sciences Kyung Hee University Yongin South Korea; ^2^ Division of Microbiology Department of Molecular Cell Biology Samsung Biomedical Research Institute Sungkyunkwan University School of Medicine Suwon South Korea; ^3^ Department of Microbial Pathogenesis Yale School of Medicine New Haven Connecticut; ^4^ Department of Molecular Bioscience College of Biomedical Science Kangwon National University Chuncheon South Korea

**Keywords:** elongation factor P, Mg^2+^ transporter, *mgtA*, translational control

## Abstract

Ribosome often stalls on mRNA sequences harboring consecutive proline codons. Elongation factor P (EF‐P) is required for the stalled ribosome to continue translation and thus the absence of EF‐P affects translation of the associated open reading frame. Here we report that EF‐P controls translation of the *mgtA* gene encoding a Mg^2+^‐transporting ATPase from the intracellualr pathogen *Salmonella enterica* serovar Typhimurium. EF‐P's effect on *mgtA* translation is dependent on the 550th and 551st proline codons in the coding region and thus substitution of those proline codons eliminates EF‐P‐mediated control of MgtA protein without affecting the Mg^2+^‐transporting activity of the *mgtA* gene. The Pro550 and Pro551‐substituted *mgtA* gene promotes *Salmonella*'s intramacrophage survival and mouse virulence, suggesting that EF‐P‐mediated translational control of the *mgtA* gene is required for *Salmonella* pathogenesis.

## 1 INTRODUCTION

If mRNA sequences harbor two or more consecutive proline codons, a ribosome often stalls at those consecutive proline codons (Doerfel et al., 2013; Peil et al., 2013; Tanner, Cariello, Woolstenhulme, Broadbent, & Buskirk, 2009; Woolstenhulme et al., 2013) due to a steric constraint imposed upon the peptide bond formation between preexisting peptidyl‐prolyl tRNA and incoming prolyl‐tRNA (Blaha, Stanley, & Steitz, 2009; Pavlov et al., 2009; Wohlgemuth, Brenner, Beringer, & Rodnina, 2008). EF‐P is a protein factor that is specifically required for the stalled ribosome to continue translation (Doerfel et al., 2013; Lassak, Wilson, & Jung, 2016; Ude et al., 2013). EF‐P promotes the peptidyl transferase activity of the ribosome by binding to E‐site within the ribosome and stabilizing the CCA end of the P‐site tRNA (Blaha et al., 2009; Huter et al., 2017). As expected from EF‐P's role in the elongation step of translation, a strain lacking EF‐P induces ribosome stalling on polyproline mRNA sequences and therefore it has an impact on expression of many genes harboring the polyproline motifs in either the coding region or the short open reading frame (ORF) within the leader RNA. If polyproline motifs are located in the coding region of a target gene, protein levels of the gene are expected to be lower in the strain lacking EF‐P than in the strain harboring EF‐P. A different scenario is also possible in bacteria if polyproline codons are located at a short ORF within the leader RNA. In this case, lack of EF‐P induces ribosome stalling at the short ORF and uncouples between translation of the short ORF and transcription of the leader RNA, which affects the formation of an attenuator stem‐loop structure. Depending on whether ribosome stalling promotes or inhibits the formation of the attenuator stem‐loop, it controls transcription elongation into the downstream genes and affects mRNA levels of those target genes. In the *mgtCBR* virulence operon from the intracellular pathogen *Salmonella enterica* serovar Typhimurium, EF‐P controls both transcription of the *mgtCBR* operon (Nam, Choi, Shin, & Lee, 2016) and translation of the *mgtB* gene encoding the MgtB Mg^2+^ transporter located within the *mgtCBR* operon (Choi, Choi, et al., 2017). At the level of transcription, EF‐P controls transcription elongation into the *mgtCBR* operon via three consecutive proline codons at *mgtP* ORF in the leader RNA. Removal of EF‐P affects ribosome stalling at *mgtP* and thus increases mRNA levels of the *mgtC* and *mgtB* genes (Choi, Choi, et al., 2017; Nam et al., 2016). And substitution of the consecutive proline codons at *mgtP* eliminates the EF‐P‐mediated increase in mRNA levels of the *mgtC* and *mgtB* genes (Choi, Choi, et al., 2017; Nam et al., 2016). In addition to EF‐P's role in transcriptional control, EF‐P also controls translation of the *mgtB* gene via two consecutive proline codons located in the *mgtB* coding region (Choi, Choi, et al., 2017). In this case, removal of EF‐P results in a decrease in protein levels of the MgtB Mg^2+^ transporter and thus substitution of those two proline codons prevents the decrease in MgtB protein levels even in the absence of EF‐P (Choi, Choi, et al., 2017). Interestingly, given that mRNA levels of EF‐P decrease during *Salmonella* infection (Nam et al., 2016), *Salmonella* is likely to produce an altered ratio of the MgtC virulence protein to the MgtB Mg^2+^ transporter (Choi, Choi, et al., 2017), which is required for *Salmonella* pathogenesis (Choi, Choi, et al., 2017; Lee & Groisman, 2010).

In addition to the *mgtB* gene in the *mgtCBR* operon, Mg^2+^ transport in *S. enterica* is mediated by two additional loci, the *mgtA* and *corA* genes (Groisman et al., 2013). Among them, the *mgtA* gene encoding the MgtA Mg^2+^ transporter is similar to the *mgtB* gene in several aspects. First, both the *mgtA* and *mgtB* genes encode the P‐type Mg^2+^ transporting ATPases (51.3% identity) that involve the influx of Mg^2+^ ions through phosphorylation of the key aspartic acid residues in the transporter proteins during each transport cycle (Groisman et al., 2013; Smith & Maguire, 1998). Second, transcription initiation of the *mgtA* and *mgtB* genes is controlled by the PhoP/PhoQ two‐component system (Soncini, Garcia Vescovi, Solomon, & Groisman, 1996), which is activated in response to low Mg^2+^ (Garcia Vescovi, Soncini, & Groisman, 1996), as well as two other signals, mildly acidic pH and antimicrobial peptides (Bader et al., 2005; Prost et al., 2007). Third, the *mgtA* gene and *mgtCBR* operon are preceded by long leader RNAs, which harbor proline codon‐rich ORFs, *mgtL* and *mgtP*, respectively (Lee & Groisman, 2012; Park, Cromie, Lee, & Groisman, 2010). The presence of proline codons in *mgtL* and *mgtP* allows EF‐P to control transcription of the associated *mgtA* gene and *mgtCBR* operon (Gall et al., 2016; Nam et al., 2016). These similarities between the *mgtA* and *mgtB* genes lead us to examine whether EF‐P controls translation of the *mgtA* Mg^2+^ transporter gene, in addition to the previously identified EF‐P's role in transcription elongation of the *mgtA* gene (Gall et al., 2016; Nam et al., 2016). In this paper, we determined that EF‐P controls translation of the *mgtA* gene via two consecutive proline codons located in the coding region. Substitution of the proline codons prevents EF‐P‐mediated translational control in MgtA protein levels without affecting the Mg^2+^‐importing activity of the MgtA protein. Moreover, removal of EF‐P's control in the *mgtA* gene promotes *Salmonella* virulence, implying that EF‐P compromises the Mg^2+^ transporting activity of the MgtA protein during *Salmonella* infection.

## 2 MATERIALS AND METHODS

### 2.1 Bacterial strains, plasmids, primers, and growth conditions

Bacterial strains, plasmids, and oligonucleotides used in this study are listed in Supporting Information Tables S1 and S2. All *S. enterica* serovar Typhimurium strains are derived from the wild‐type strain 14028s (Fields, Swanson, Haidaris, & Heffron, 1986) using one‐step gene inactivation method (Datsenko & Wanner, 2000) and were constructed by phage P22‐mediated transductions (Davis, Bolstein, & Roth, 1980). Bacteria were grown at 37°C in Luria–Bertani broth (LB), N‐minimal media (pH 7.7) (Snavely, Miller, & Maguire, 1991) supplemented with 0.1% casamino acids, 38 mM glycerol and the indicated concentrations of MgCl_2_. Ampicillin was used at 50 μg/ml, chloramphenicol at 25 μg/ml, kanamycin at 50 μg/ml, or tetracycline at 10 μg/ml, respectively.

### 2.2 RNA extraction and quantitative real‐time polymerase chain reaction (RT‐PCR)

Total RNA was isolated using RNeasy Kit (Qiagen) according to the manufacturer's instructions. A quantity 500 ng to 1 μg of the purified RNA was synthesized to cDNA, using PrimeScript^TM^ RT reagent kit (TakaRa). The mRNA levels of the *mgtA* and *mgtB* gene were measured by SYBR Green PCR Master Mix (TOYOBO) and appropriate primers (*mgtA*: 4308/4309, *mgtB*: 7763/7764, and *rrsH*: 6970/6971) and monitored, using a 7500 Fast Real‐Time PCR system (Applied Biosystems, Foster City).

### 2.3 Western blot analysis

Cells were grown for 5 hr or 6 hr in 35 ml of N‐minimal medium‐containing 0.01 mM or 10 mM Mg^2+^. Cells were normalized by measuring optical density at 600 nm (OD_600_) and pelleted by centrifugation. Crude extracts were prepared in TBS (Tris‐buffered saline) buffer by sonication and analyzed as described (Lee & Groisman, 2010). The intensity of bands in the blots was quantified, using Image J software. The data are representative of two independent experiments, which gave similar results.

### 2.4 Measuring growth of strains lacking Mg^2+^ transporters

To address whether the *mgtA* proline substitution mutant carries the Mg^2+^‐transporting activity in a strain lacking two other Mg^2+^ transporters, MgtB, and CorA, cells were grown in N‐minimal medium containing 0.01 mM or 100 mM Mg^2+^. Growth was determined at 37°C for 7 hr in a 96‐well plate with orbital shaking and absorbance was measured at OD_600_ every 2.5 min, using Synergy H1 (BioTek).

### 2.5 *Salmonella's* survival inside macrophages

Intramacrophage survival assays were conducted with the macrophage‐like cell line J774 A.1 as described (Blanc‐Potard & Groisman, 1997).

### 2.6 Mouse virulence assays

Six‐ to eight‐week‐old female C3H/HeN mice were inoculated intraperitoneally with ~10^3^ colony‐forming units (CFU) of *Salmonella* strains. Mouse survival was followed for 21 days. Virulence assays were conducted twice with similar outcomes, and data correspond to groups of five mice. All procedures were performed according to approved protocols by the Institutional Animal Care and Use Committee from Kangwon National University.

### 2.7 Construction of a strain with the chromosomal *mgtA* deletion

The one‐step gene inactivation method (Datsenko & Wanner, 2000) was used for the chromosomal *mgtA* gene deletion. For construction of the *mgtA* deletion strain, the Km^R^ cassette from plasmid pKD4 (Datsenko & Wanner, 2000) was amplified using primers DE‐*mgtA*‐F and DE‐*mgtA*‐R and integrated into 14028s chromosome to create EN394 (*mgtA*::Km^R^). The Km^R^ cassette was removed using plasmid pCP20 (Datsenko & Wanner, 2000) to generate EN396 (*mgtA*).

### 2.8 Construction of chromosomal mutant strains with the *mgtA* proline codons substituted by alanine codons

We substituted consecutive proline codons in the *mgtA* coding region to alanine codons using the fusaric acid‐based counterselection method (Lee & Groisman, 2010). First, we introduced Tet^R^ cassettes in two different regions of the *mgtA* gene as follows: We generated PCR products harboring *tetRA* genes using primers KHU546/KHU547 (for 39th and 40th proline codons), KHU548/KHU549 (for 550th and 551st proline codons substitution in the *mgtA*‐HA background) and KHU602/KHU603 (for 550th and 551st proline codons substitution in the wild‐type background) and MS7953s genomic DNA as a template. The PCR products were purified using a QIAquick PCR purification kit (QIAGEN) and used to electroporate EN336 (*mgtA*‐HA) or 14028s strain containing plasmid pKD46 (Datsenko & Wanner, 2000). The resulting *mgtA*(up)‐HA::*tetRA* (EN921), *mgtA*(down)‐HA::*tetRA* (EN922) and *mgtA*(down)::*tetRA* (EN981) strains containing plasmid pKD46 were kept at 30°C for the following step. Then, we replaced the *tetRA* cassettes by DNA fragments carrying proline to alanine substitutions in *mgtA* at positions 39 and 40 or 550 and 551. The DNA fragments were prepared by a two‐step PCR process. For the first PCR, we used two sets of primer pairs KHU550/KHU553 and KHU552/KHU551 (for 39th and 40th proline codon), KHU554/KHU557 and KHU556/KHU555 (for 550th and 551st proline codon substitution in the *mgtA*‐HA background) and KHU606/KHU557 and KHU556/KHU607 (for 550th and 551st proline codon substitution in the wild‐type background) and 14028s genomic DNA as template. For the second PCR, we mixed the two PCR products from the first PCR as templates and amplified DNA fragments using primers KHU550/KHU551 (for 39th and 40th proline codon substitution) and KHU554/KHU555 (for 550th and 551st proline codon substitution in the *mgtA*‐HA background) and KHU606/KHU607 (for 550th and 551st proline codon substitution in the wild‐type background). The resulting PCR products were purified and integrated into the EN921, EN922 and EN981 chromosomes and selected against tetracycline resistance with media containing fusaric acid (Lee & Groisman, 2010) to generate EN932 (*mgtA* ^Pro 39,40 Ala^‐HA), EN933 (*mgtA* ^Pro 550,551 Ala^‐HA) and EN982 (*mgtA* ^Pro 550,551 Ala^), tetracycline‐sensitive, ampicillin‐sensitive chromosomal mutants, respectively. The presence of the expected nucleotide substitutions was verified by DNA sequencing. A P22 phage lysate grown in strain DN337 (*efp*::Cm^R^) was used to transduce strains EN932, and EN933 *Salmonella* selecting for chloramphenicol resistance to generate EN940 (*mgtA* ^Pro 39,40 Ala^‐HA, *efp*::Cm^R^), and EN941 (*mgtA* ^Pro 550,551 Ala^‐HA, *efp*::Cm^R^), respectively.

## 3 RESULTS

### 3.1 *efp* deletion increases *mgtA* mRNA levels in a manner dependent on the interspersed proline codons at *mgtL* located in the leader region

**Figure 1:**
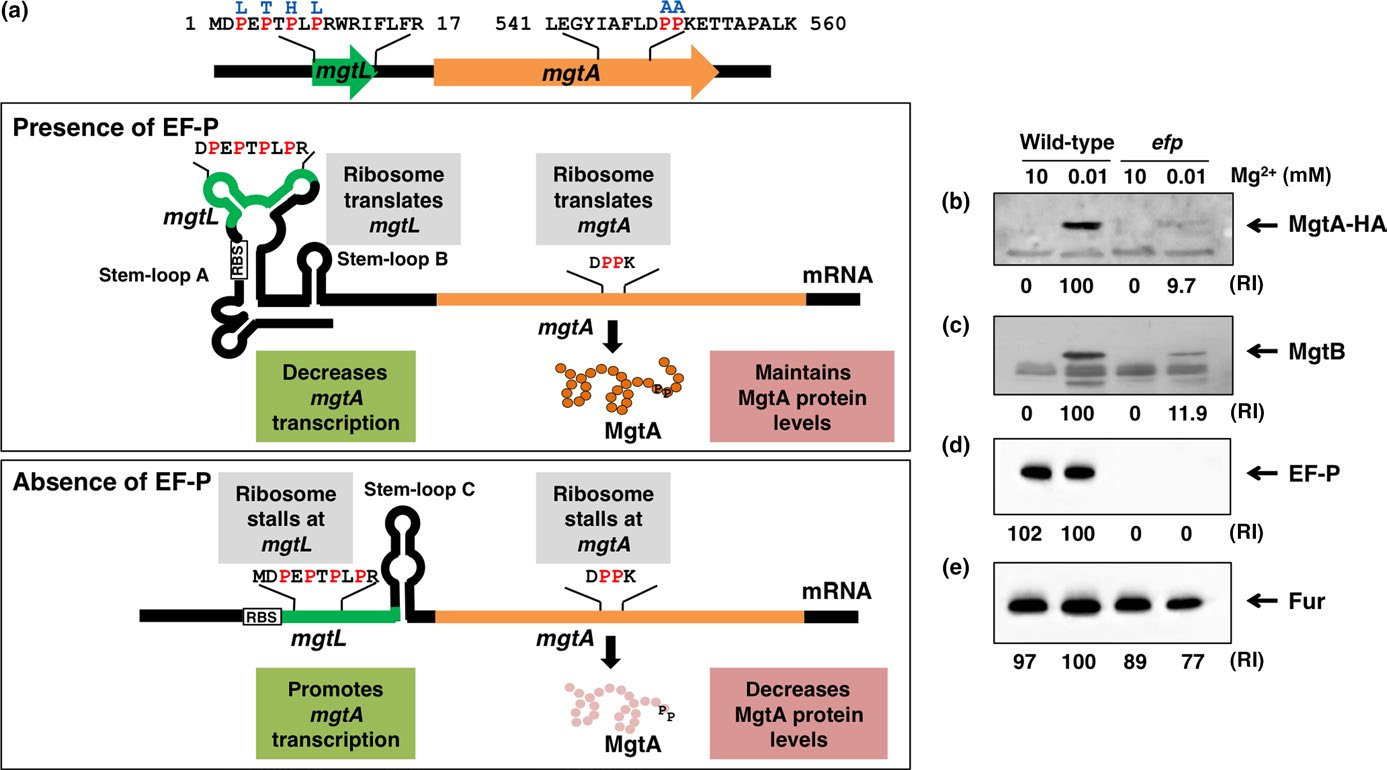
Regulation of the *mgtA* gene encoding the MgtA Mg^2+^ transporter by EF‐P. (a) The *mgtA* gene has the 264 nt‐long leader region and harbors a short ORF (*mgtL*: encoded by nucleotide 71–124) within the leader region that controls transcription elongation of the associated coding region in response to proline‐tRNA ^pro^ levels. The *mgtL* have conserved four proline codons with an alternating arrangement. Also, the *mgtA* gene has consecutive proline codons at positions 550 and 551 in the coding region. When EF‐P is present, ribosomes translate *mgtL* and allow the leader to form stem‐loop B, decreasing *mgtA* mRNA levels. However, EF‐P maintains MgtA protein levels by helping ribosomes to translate the *mgtA* gene. By contrast, when EF‐P is absent, ribosomes stall at proline codons in *mgtL* and enable the formation of stem‐loop C, resulting in an increase of *mgtA* transcripts. Interestingly, even though *mgtA* mRNA levels are elevated, MgtA protein levels decrease because ribosomes stall proline codons in the *mgtA* coding region due to lack of EF‐P. Amino acid substitutions used in this study are indicated in blue. (b–e) *Salmonella* lacking EF‐P decreases MgtA protein levels. Western blot analysis of crude extracts prepared from strains with the C‐terminally HA‐tagged *mgtA* gene in either the wild‐type (EN336) or *efp* deletion mutant *Salmonella* (EN897). Blots were probed with anti‐HA (b), anti‐MgtB (c), anti‐EF‐P (d), or anti‐Fur (e) antibodies to detect MgtA‐HA, MgtB, EF‐P, and Fur proteins, respectively. Bacteria were grown for 5 hr in N‐minimal media containing 10 mM or 0.01 mM Mg^2+^ as described in Materials and Methods. Numbers below the blots correspond to the relative amount of proteins when the amount present in the wild‐type strain at low Mg^2+^ is set to 100. RI represents relative intensity

The 264‐nt long leader region controls transcription elongation of the *mgtA* gene by a transcription attenuation mechanism, whose critical step is the formation of the attenuator stem‐loop (stem‐loop B, Figure 1a). The formation of stem‐loop B is determined by a degree of the coupling/uncoupling between transcription of the leader region and translation of *mgtL* located within the leader region of the *mgtA* gene (Park et al., 2010). Therefore, any conditions that slow down translation of *mgtL* ORF could uncouple transcription of the leader region and translation of *mgtL* and inhibit the formation of stem‐loop B, resulting in an increase in transcription of the *mgtA* coding region (Figure 1a). The presence of alternating proline codons at 3rd, 5th, 7th, and 9th positions in *mgtL* raised the possibility that *efp* deletion affects ribosome stalling at *mgtL* and thus increases *mgtA* transcription. To test this possibility, we measured the effect of *efp* deletion on mRNA levels of the *mgtA* gene in the wild‐type or a derivative *Salmonella* where we substituted the *mgtL* proline codons by other codons (Leu, Thr, His, Leu) (Please note that the *mgtL* proline substitution eliminates proline codons but retains a base‐pairing required for the Mg^2+^‐sensing riboswitch (Park et al., 2010; Cromie et al., 2006)). As expected, the *efp* deletion mutant increased mRNA levels of the *mgtA* gene by ~12 fold (Gall et al., 2016; Nam et al., 2016) (Supporting Information Figure S1A). The increase in *mgtA* mRNA levels is dependent on interspersed proline codons at *mgtL* because the elevation of *mgtA* mRNA levels was not detected in the *mgtL* derivative with the Pro substitution (Supporting Information Figure S1A). This indicates that *efp* deletion affects ribosome stalling at those proline codons in *mgtL* even though they are not consecutive and allows the leader to increase *mgtA* mRNA levels. Control experiments proved that the Pro substitution at *mgtL* did not affect the expression behaviors of *mgtB* and *efp* genes (Supporting Information Figure S1B and C).

### 3.2 *Salmonella* lacking EF‐P decreases MgtA protein levels compared to *Salmonella* producing EF‐P in low Mg^2+^

**Figure 2:**
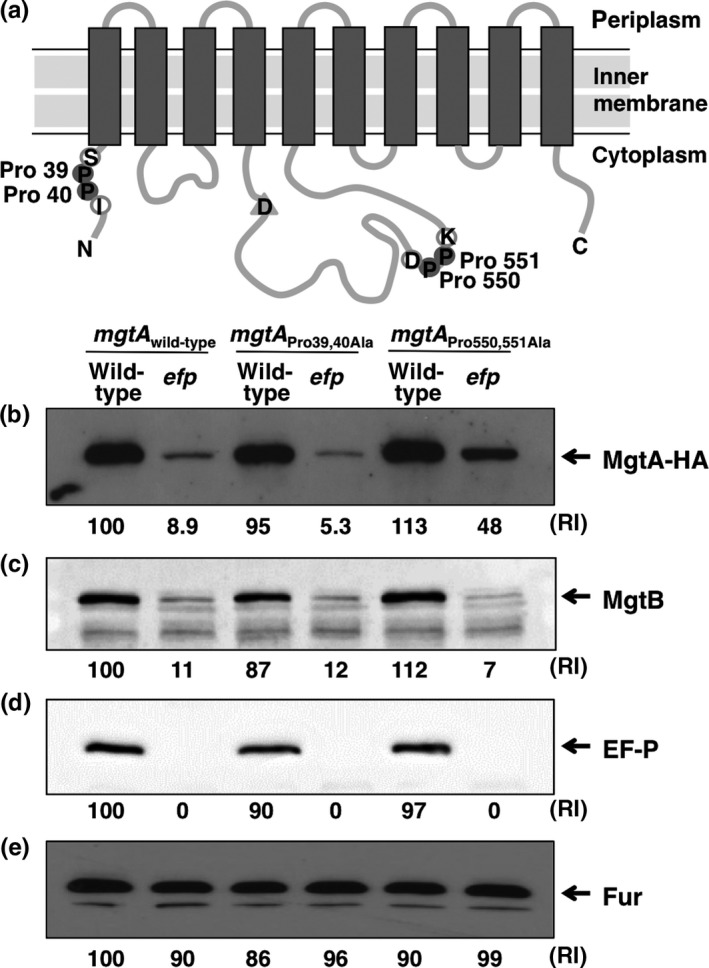
EF‐P is required for MgtA production in a manner dependent on Pro550 and Pro551 codons. (a) Schematic diagram of the MgtA protein. The locations of proline codons (gray closed circles) and neighboring codons (gray open circles) used in this work are indicated. Asp377 required for phosphorylation (triangle) is indicated. (B–E) Western blot analysis of crude extracts prepared from a strain with the wild‐type *mgtA*‐HA gene (EN336), the *efp* deletion mutant (EN897), an *mgtA‐* HA derivative with the Pro39 and Pro40 codons substituted by Ala codons (EN932), a mutant with both the Pro39 and Pro40 substitution and the *efp* deletion (EN940), an *mgtA*‐HA derivative with the Pro550 and Pro551 codons substituted by Ala codons (EN933), or a mutant with both the Pro550 and Pro551 substitution and the *efp* deletion (EN941). Blots were probed with anti‐HA (b), anti‐MgtB (c), anti‐EF‐P (d), or anti‐Fur (e) antibodies to detect MgtA‐HA, MgtB, EF‐P, and Fur proteins, respectively. Bacteria were grown for 5 hr in N‐minimal media containing 0.01 mM Mg^2+^ as described in Materials and Methods. Numbers below the blots correspond to the relative amount of proteins when the amount present in the wild‐type strain at low Mg^2+^ is set to 100. RI represents relative intensity

In addition to the previous finding that EF‐P controls transcription of the *mgtA* gene by the leader RNA (Gall et al., 2016; Nam et al., 2016) (Supporting Information Figure S1), we suspected that EF‐P might control translation of *mgtA* gene based on following: First, MgtA protein harbors two polyproline motifs at positions 39 and 40, and 550 and 551 (Supporting Information Figure S2A and B). The location of the consecutive proline codons and the sequences neighboring the proline codons are well conserved with those in the MgtB protein, which is another Mg^2+^‐importing transporter in *Salmonella* (Figure 2a and Supporting Information Figure S2A and B) (Snavely et al., 1991). Second, our previous study identified that EF‐P controls translation of the *mgtB* gene via one of those consecutive proline codons (Choi, Choi, et al., 2017). To explore this, we created chromosomal mutant strains with the C‐terminally HA‐tagged *mgtA* gene in the wild‐type or *efp* mutant *Salmonella*. Because transcription of the *mgtA* gene is controlled by the PhoP/PhoQ two‐component regulatory system (Soncini et al., 1996), we measured MgtA protein levels in a PhoP‐inducing (0.01 mM Mg^2+^) or PhoP‐repressing (10 mM Mg^2+^) condition. Wild‐type *Salmonella* increased MgtA protein production in low Mg^2+^ media, whereas *Salmonella* lacking EF‐P produced lower levels of the MgtA protein compared to those of the wild‐type in the same media (Figure 1b). And, this is similar to what we observed in MgtB protein levels (Figure 1c) (Choi, Choi, et al., 2017), indicating that EF‐P controls translation of both the *mgtA* and *mgtB* genes encoding the MgtA and MgtB Mg^2+^ transporters respectively. Control experiments proved as follows: Both MgtA and MgtB proteins were not detected in high Mg^2+^ media (Figure 1b,c). And *efp* deletion abolished EF‐P protein production (Figure 1d) but had no effect on Fur protein levels (Figure 1e).

### 3.3 Consecutive proline codons at positions 550 and 551 in the *mgtA* gene are required for EF‐P‐mediated translational control

If EF‐P controls translation of the *mgtA* gene via consecutive proline codons, we wondered which proline codons are required for EF‐P‐mediated MgtA translational control. To investigate this, we created chromosomal mutants where consecutive proline codons at positions 39 and 40 or 550 and 551 in the C‐terminally HA‐tagged *mgtA* gene were substituted by alanine codons in either the wild‐type or *efp* deletion mutant background (Figure 2a). The *efp* deletion decreased MgtA protein levels in a strain with the wild‐type *mgtA* gene in low Mg^2+^ (Figure 2b). However, the proline substitution at positions 550 and 551 in the *mgtA* coding region partially restored production of the MgtA protein in the *efp* deletion mutant (Figure 2b), demonstrating that consecutive proline codons at positions 550 and 551 are required for EF‐P‐mediated *mgtA* translational control. By contrast, the Pro39 and Pro40 substitution did not restore MgtA protein production but further decreased MgtA protein levels in the *efp* deletion mutant (Figure 2b). Control experiments indicated that the proline substitutions of the *mgtA* gene did not affect the expression patterns of the MgtB, EF‐P, and Fur proteins (Figure 2c–e).

### 3.4 The *mgtA* with the Pro550 and Pro551 substitution has no defect of the Mg^2+^‐ transporting activity in the strain lacking other Mg^2+^ transporters

**Figure 3:**
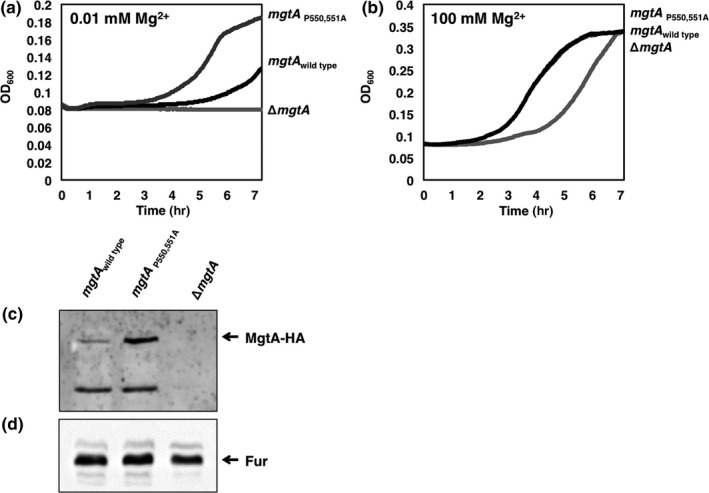
MgtA with the Pro 550, 551Ala substitution in the *mgtA* gene supports growth in low Mg^2+^ in a strain lacking other Mg^2+^ transporters. (a–b) Growth curves of a strain with the wild‐type *mgtA* gene (EN977), the *mgtA* derivative with the Pro 550, 551 codons substituted by Ala codons (EN998), or the *mgtA* deletion (EL498) in a genetic background where both *corA* and *mgtB* genes are deleted. Bacteria were grown in N‐minimal medium containing 0.01 mM (a) or 100 mM Mg^2+^ (b) at 37°C for 7 hr in a 96‐well plate with orbital shaking and measured absorbance at OD_600_ every 2.5 min. (c–d) Western blot Analysis of crude extracts prepared from *Salmonella* strains listed above. Blots were probed with anti‐HA (c) and anti‐Fur (d) antibodies to detect MgtA and Fur proteins, respectively. Bacteria were grown for 6 hr in N‐minimal medium containing 0.01 mM Mg^2+^

Next, we asked whether the Pro550 and Pro551 substitution has an impact on the Mg^2+^‐importing activity of the MgtA protein because the Pro550 and Pro551 residues and the phosphorylated Asp377 residue are located in the same large cytoplasmic loop between 4th and 5th transmembrane helices (Figure 2a). To exclude a Mg^2+^‐transporting activity from other Mg^2+^ transporters, we created strains deleted both the *mgtB* and *corA* genes encoding the MgtB and CorA Mg^2+^ transporters in *Salmonella* strains with the wild‐type *mgtA* gene or the *mgtA* derivative with the Pro550 and Pro551 substitution. Then, we tested whether they could support growth in N‐minimal media depleting Mg^2+^. The proline substitution at positions 550 and 551 in the *mgtA* could support growth of the strain lacking other Mg^2+^ transporters (Figure 3a), suggesting that the MgtA protein with the proline substitution retains the ability to import Mg^2+^. It is interesting to note that the proline substitution mutant grew faster than *Salmonella* carrying the wild‐type *mgtA* gene because MgtA protein levels in the proline substitution mutant are higher than those in the wild‐type (Figure 3c). By contrast, an introduction of the *mgtA* deletion completely abolished growth in low Mg^2+^ media (Figure 3a), further supporting that the ability to grow in low Mg^2+^ media is mediated by the presence of the functional *mgtA* gene in the strains lacking other Mg^2+^ transporters. As a control, supplementation of 100 mM Mg^2+^ supports the growth of all strains tested (Figure 3b) (Hmiel, Snavely, Florer, Maguire, & Miller, 1989).

### 3.5 The *mgtA* with the Pro550 and Pro551 substitution increases *Salmonella's* survival inside macrophages and virulence in mice

**Figure 4:**
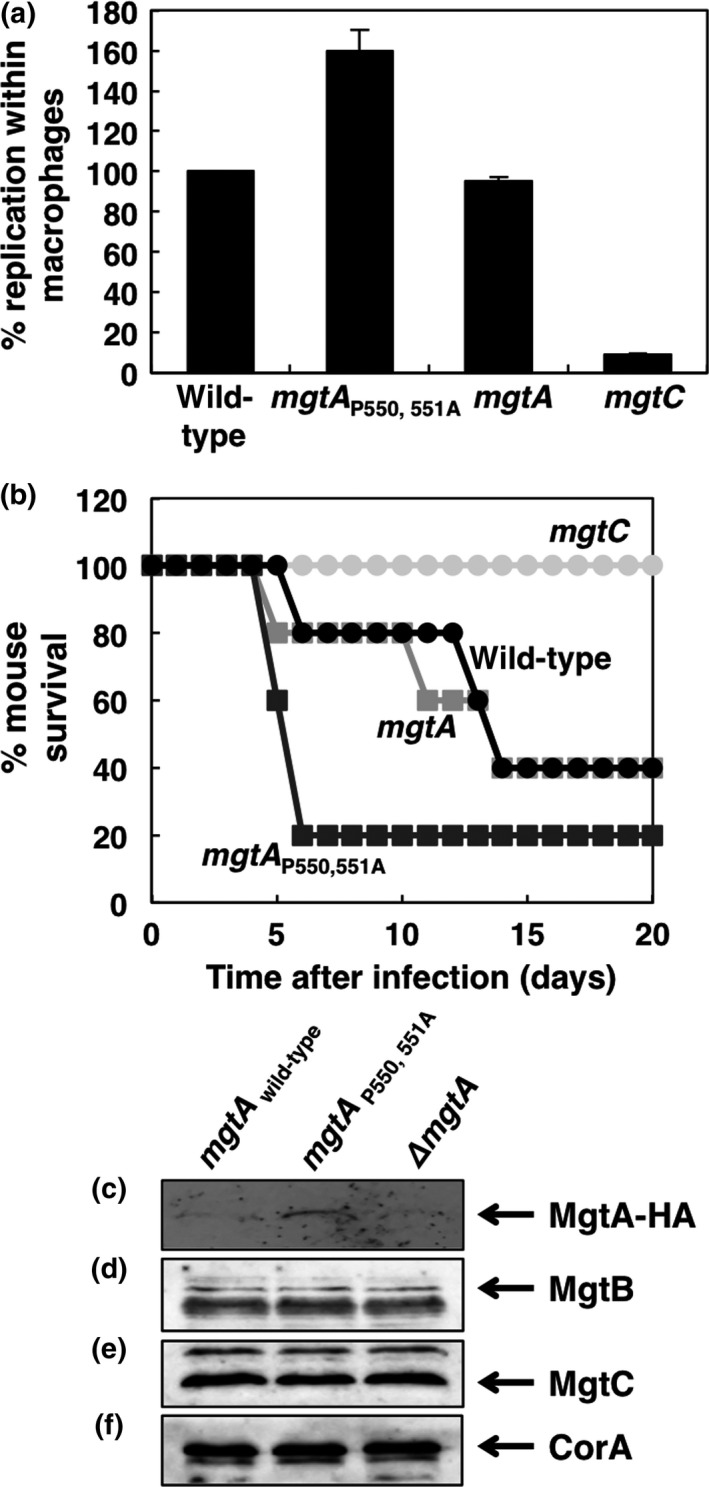
The *mgtA* Pro 550, 551 to Ala substitution promotes *Salmonella*'s survival inside macrophages and virulence in mice. (a) Replication inside J774 A.1 macrophages of wild‐type (14028s), the *mgtA* derivative with Pro codons replaced by Ala codons (EN982), the *mgtA* deletion mutant (EN396), or an *mgtC* deletion mutant (EL4) *Salmonella* at 21 hr after infection. (b) Survival of C3H/HeN mice inoculated intraperitoneally with ~2000 colony forming units of the *Salmonella* strains listed above. (c–f). Western blot analysis of crude extracts prepared from a strain with the wild‐type *mgtA*‐HA gene (EN336), the *mgtA‐* HA derivative with the Pro550 and Pro551 codons substituted by Ala codons (EN933), or the *mgtA* deletion mutant (EN396) inside macrophages at 9 hr after infection. Blots were probed with anti‐HA (c), anti‐MgtB (d), anti‐MgtC (e), or anti‐CorA (e) antibodies to detect MgtA‐HA, MgtB, MgtC, and CorA proteins, respectively

Because EF‐P mRNA levels decrease during *Salmonella* infection (Nam et al., 2016) and also because the Pro550 and Pro551 substitution increases MgtA protein levels in the *efp* deletion mutant (Figure 2b), we expected that the Pro550 and Pro551 substitution in the *mgtA* gene might increase *Salmonella*'s survival inside macrophages by promoting the MgtA Mg^2+^‐transporting activity. Indeed, the *Salmonella* strain with the Pro550 and Pro551‐substituted *mgtA* gene increased survival inside macrophages by ~160% compared to those of the wild‐type (Figure 4a). This is due to that the Pro550 and Pro551 substitution increased MgtA protein levels inside macrophages (Figure 4c). As described previously (Blanc‐Potard & Groisman, 1997), the *mgtA* deletion mutant had no apparent effect on intramacrophage survival (Figure 4a). As control experiments, the *mgtC* mutant showed a severe defect in macrophage survival (Blanc‐Potard & Groisman, 1997; Lee, Pontes, & Groisman, 2013)(Figure 4a) and the Pro550 and Pro551 substitution in the *mgtA* gene did not affect protein levels of MgtB, MgtC, and CorA proteins (Figure 4d–f).

Similarly, the Pro550 and Pro551 substitution in the *mgtA* gene promoted virulence in mice when mice were injected *Salmonella* strains intraperitoneally (Figure 4b). Therefore, it indicates that EF‐P‐mediated translational control of the MgtA protein is required for *Salmonella*'s pathogenicity during infection.

## 4 DISCUSSION

Here, we showed a new example of EF‐P's role in translation of the *mgtA* Mg^2+^ transporter gene. We determined that lack of EF‐P decreases MgtA protein levels and the decrease is mediated by the 550th and 551st proline codons in the *mgtA* coding region (Figures 1 and 2). Substitution of the Pro550 and Pro551 codons prevents the EF‐P‐mediated decrease of MgtA protein levels, thereby producing significant levels of the MgtA protein even in *Salmonella* lacking EF‐P (Figure 2). This property of the Pro550 and Pro551‐substituted *mgtA* gene also influences *Salmonella*'s pathogenicity (Figure 4) because *Salmonella* decreases mRNA levels of EF‐P inside macrophages (Nam et al., 2016). Accordingly, *Salmonella* with the Pro550 and Pro551‐substituted *mgtA* enhances survival inside macrophages and virulence in mice by increasing the amounts of the MgtA Mg^2+^ transporter (Figure 4). Given that the *mgtA* Pro550 and Pro551 substitution renders *Salmonella* hypervirulent, it reflects that wild‐type *Salmonella* needs to compromise the Mg^2+^‐transporting activity of the MgtA protein during infection. And, this is further supported by the previous finding that EF‐P mediates the decrease in the protein levels of another MgtB Mg^2+^ transporter during infection (Choi, Choi, et al., 2017).

It is interesting to note that EF‐P controls translation of both the *mgtA* and *mgtB* genes encoding the Mg^2+^‐transporting ATPases via two consecutive proline codons in the respective coding regions. Moreover, elimination of EF‐P's control from either the *mgtA* or *mgtB* genes by substituting the proline codons renders *Salmonella* hypervirulent, reinforcing the notion that EF‐P limits Mg^2+^ transport during infection by a similar regulatory mechanism imposed upon translation of the *mgtA* and *mgtB* Mg^2+^ transporter genes. By decreasing Mg^2+^ uptake during infection, *Salmonella* seems to coordinate intracellular Mg^2+^ levels with intracellular ATP levels, which are also limited by the MgtC virulence protein within a macrophage phagosome (Lee et al., 2013; Pontes, Lee, Choi, & Groisman, 2015).

One might wonder why *Salmonella* increases *mgtA* mRNA levels via the proline codons of *mgtL* in the leader RNA despite decreasing MgtA protein levels via the proline codons in the *mgtA* coding region when *Salmonella* is inside macrophages and thus decreases *efp* mRNA levels (Figure 1). Does it have a potential role for the *mgtA* transcripts independent of producing the MgtA proteins? In addition to a previous example (Loh et al., 2009), a recent study may provide a clue for a potential role for the *mgtA* transcripts because one of the small leader transcripts of the *mgtCBR* operon is highly expressed inside macrophages and functions as a *trans*‐acting riboregulator for downregulating synthesis of the *Salmonella* flagellin protein during infection (Choi, Han, Cho, Nam, & Lee, 2017). Therefore, a future exploration seeking a potential role(s) of the *mgtA* transcripts independent of its protein functions is expected.

A previous systemic approach found that lack of EF‐P induces ribosome pausing the consecutive motif (VPPS) located near the N‐terminus of the MgtA Mg^2+^ transporter from *Escherichia coli* (Elgamal et al., 2014). Based on 90% identity between the *mgtA* genes from *E. coli* and *S. enterica*, the identified pause sequence corresponds to the sequence including Pro39 and Pro40 (IPPS) (Figure S2). Even though they are highly similar to each other, the Pro39 and Pro40 substitution has no impact on MgtA protein levels upon *efp* deletion whereas the Pro550 and Pro551 substitution has. Therefore, it needs further attention to understand additional information to explain the discrepancy between the EF‐P‐mediated ribosomal pause site detected on ribosomal profiling data and the substitution effect on production of the MgtA proteins detected the strain lacking EF‐P.

## CONFLICT OF INTEREST

The authors declare no conflict of interest.
