# Supplementary figures and images for: Elongation factor P controls translation of the mgtA gene encoding a Mg2+ transporter during Salmonella infection
Source: Microbiologyopen. 2018 Jun 27;8(4):e00680. doi: 10.1002/mbo3.680 (PMC6460261; doi:10.1002/mbo3.680)

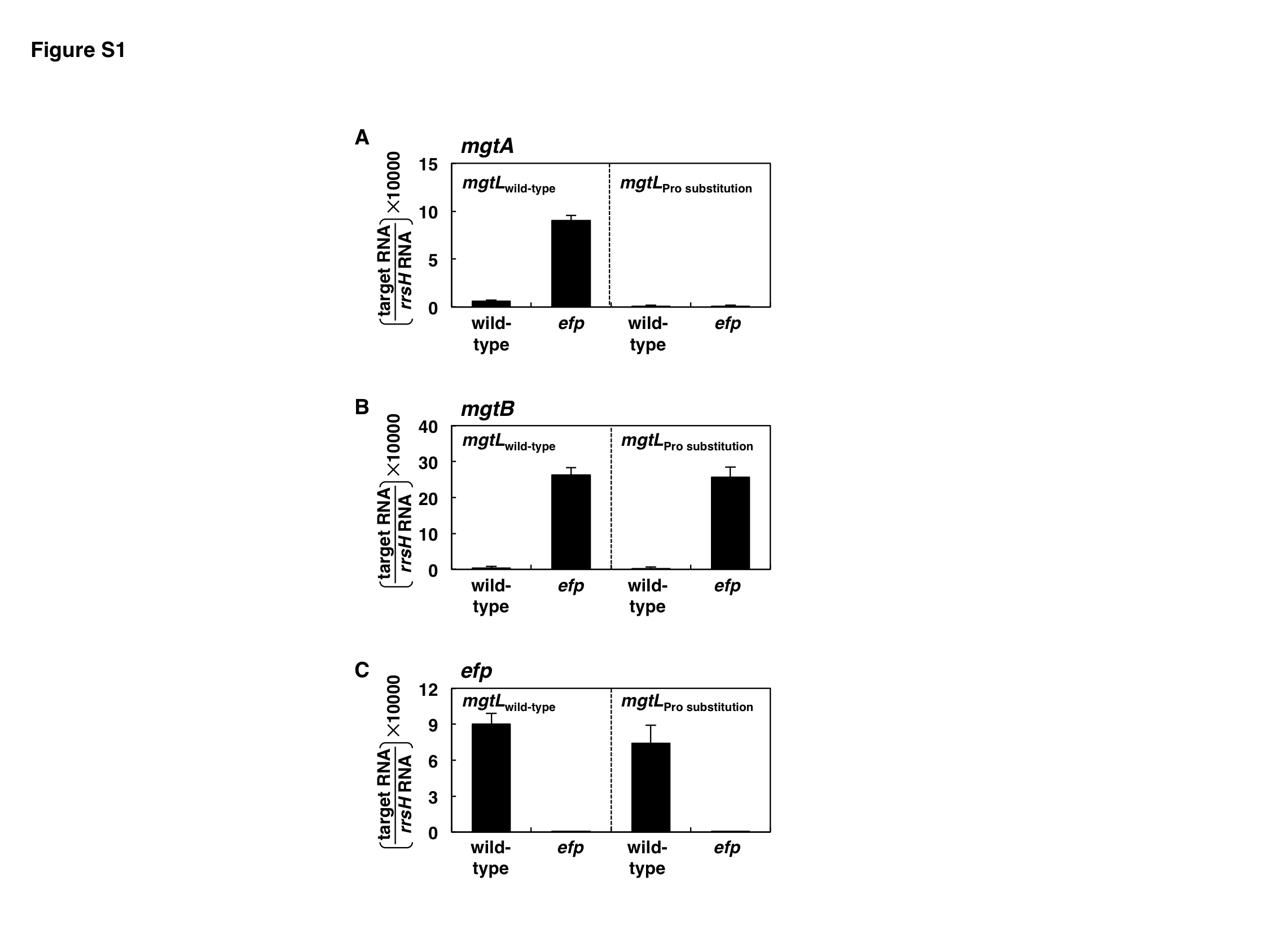

Supplement: Supplementary file 1 [file MBO3-8-e00680-s001.tiff]

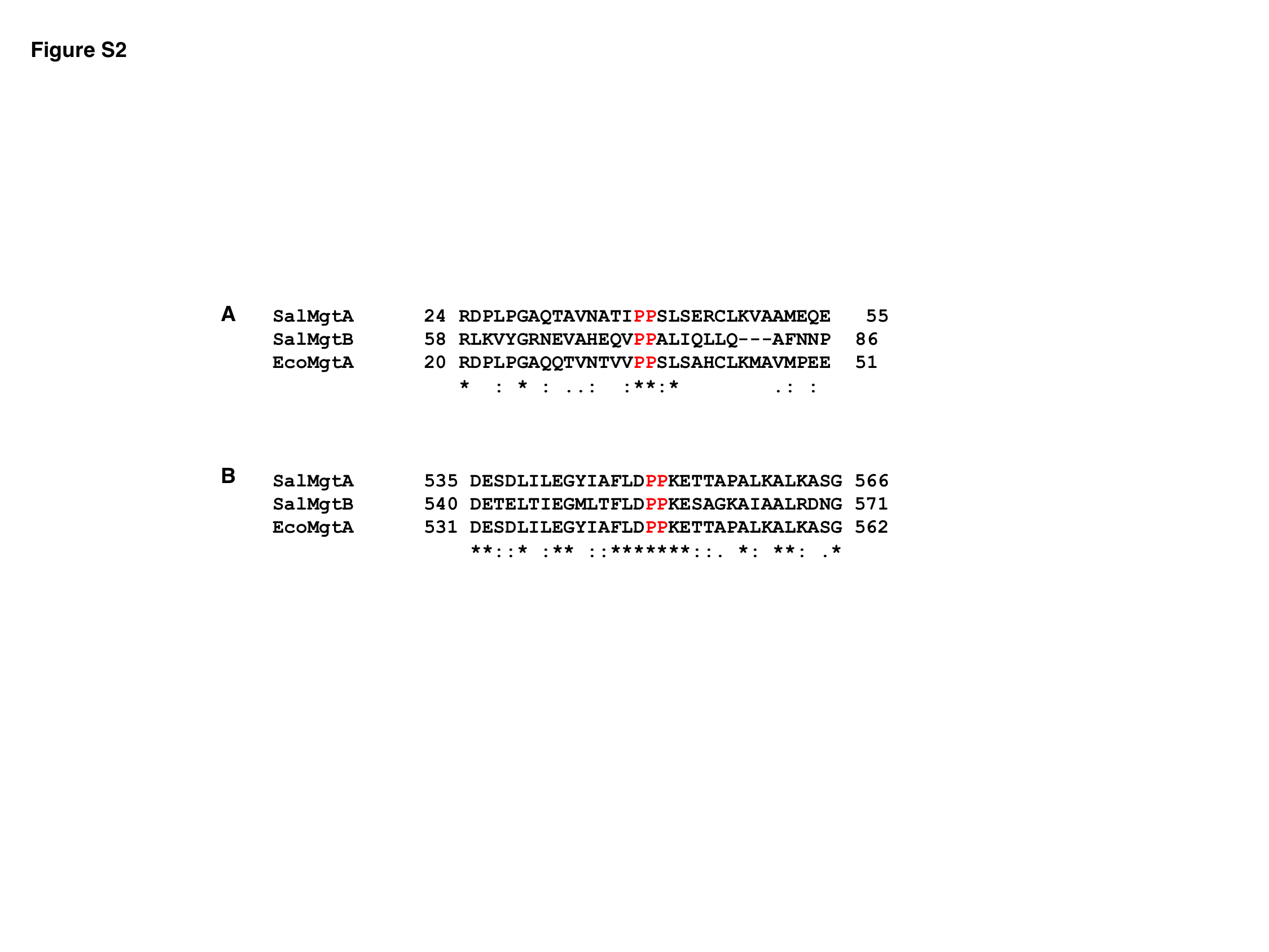

Supplement: Supplementary file 2 [file MBO3-8-e00680-s002.tiff]
