# Supplementary material for: Elongation factor P controls translation of the mgtA gene encoding a Mg2+ transporter during Salmonella infection
Source: Microbiologyopen. 2018 Jun 27;8(4):e00680. doi: 10.1002/mbo3.680 (PMC6460261; doi:10.1002/mbo3.680)
Supplement: Supplementary file 3 [file MBO3-8-e00680-s003.docx]

**Table S1. Bacterial strains and plasmids used in this study**

| **Strain or plasmid** | **Description** | **Reference or source** |
| --- | --- | --- |
| ***S. enteric* serovar Typhimurium** |  |  |
| 14028s | wild-type | ([Fields *et al.*, 1986](#_ENREF_6)) |
| MS7953s | *phoP*7953::Tn10 | ([Fields *et al.*, 1989](#_ENREF_5)) |
| YS166 | *corA*::Cm^R^ | ([Choi *et al.*, 2017](#_ENREF_1)) |
| YS957 | *up-mgtA*::Cm^R^ | ([Cromie *et al.*, 2006](#_ENREF_3)) |
| DN337 | *efp*::Cm^R^ | ([Nam *et al.*, 2016](#_ENREF_8)) |
| DN371 | *efp, up-mgtA*::Cm^R^ | ([Nam *et al.*, 2016](#_ENREF_8)) |
| DN373 | *efp, up-mgtA*::Cm^R^*, mgtL Pro 3,5,7,9* | ([Nam *et al.*, 2016](#_ENREF_8)) |
| EG19870 | *up-mgtA*::Cm^R^*, mgtL Pro 3,5,7,9* | ([Park *et al.*, 2010](#_ENREF_9)) |
| EN336 | *mgtA*-HA | ([Choi *et al.*, 2012](#_ENREF_2)) |
| EN897 | *mgtA*-HA, *efp*::Cm^R^ | This study |
| EN932 | *mgtA* ^Pro 39,40 Ala^-HA | This study |
| EN940 | *mgtA* ^Pro 39,40 Ala^-HA, *efp*::Cm^R^ | This study |
| EN933 | *mgtA* ^Pro 550,551 Ala^-HA | This study |
| EN941 | *mgtA* ^Pro 550,551 Ala^-HA, *efp*::Cm^R^ | This study |
| EN921 | *mgtA*(up)-HA::*tetRA* | This study |
| EN922 | *mgtA*(down)-HA::*tetRA* | This study |
| EN394 | *mgtA*::Km^R^ | This study |
| EN396 | *mgtA* | This study |
| EL4 | *mgtC* | ([Lee *et al.*, 2013](#_ENREF_7)) |
| EL498 | *corA*::Cm^R^*, mgtA*::MudJ*, mgtB* | ([Choi *et al.*, 2017](#_ENREF_1)) |
| EN977 | *corA*::Cm^R^*, mgtB* | This study |
| EN981 | *mgtA*(down)::*tetRA* | This study |
| EN982 | *mgtA* ^Pro 550,551 Ala^ | This study |
| EN998 | *mgtA* ^Pro 550,551 Ala^, *corA*::Cm^R^*, mgtB* | This study |
|  |  |  |
| **plasmids** |  |  |
| pKD4 | repR_6Kγ_ Ap^R^ FRT Km^R^ FRT | ([Datsenko & Wanner, 2000](#_ENREF_4)) |
| pKD46 | rep_pSC101_^ts^ Ap^R^ P*_araBAD_* γ β exo | ([Datsenko & Wanner, 2000](#_ENREF_4)) |
| pCP20 | rep_pSC101_^ts^ Ap^R^ Cm^R^ *cI857* λP_R_*flp* | ([Datsenko & Wanner, 2000](#_ENREF_4)) |
|  |  |  |

**Table S2. Oligonucleotides used in this study**

| **Primers** | **Sequences (5’ to 3’)** |
| --- | --- |
| DE-*mgtA*-F | AGAATTTTCTGCGCCTGACTTCGGCGCGGAGGGATTACCTTGTAGGCTGGAGCTGCTTCG |
| DE-*mgtA*-R | TCGGGGATTAAGCACGCTGGCGAATCCCCGACGAAAGTGTCATATGAATATCCTCCTTAG |
| 5-*mgtA*-F | AGAATTTTCTGCGCCTGACTTCGGCGC |
| 3-*mgtA*-R | GGATTAAGCACGCTGGCGAATCCCCGA |
| KHU546 | AATGGGCAAAGTCTGGTTTATCGTTGGTTTAATTACGTAATTAAGACCCACTTTCACATTTAAG |
| KHU547 | CCGCCGCCAGCTTAATAATATCGCCGGGCACCAGTTGATCCTAAGCACTTGTCTCCTGTTTAC |
| KHU548 | ATATTTCTGGTAAGCCCAGCGAGCATGTACTGCATTGCGCTTAAGACCCACTTTCACATTTAAG |
| KHU549 | GACGACCTTCAATGACGCCTTCTTCCAGCACCATCAGGCTCTAAGCACTTGTCTCCTGTTTAC |
| KHU550 | GGTTGATTTCCCTACGCCGCTCA |
| KHU551 | TTTGCCGGGGCTCACGCGTCGCG |
| KHU552 | ATGCGACTATCGCCGCCTCGCTGAG |
| KHU553 | CTCAGCGAGGCGGCGATAGTCGCAT |
| KHU554 | CGAAACAGAAAGTTATCGTGAAG |
| KHU555 | GCAAGTGCAGCGGCAGCATTGGC |
| KHU556 | TTTCTCGATGCCGCCAAAGAGACC |
| KHU557 | GGTCTCTTTGGCGGCATCGAGAAA |
| KHU602 | GCGTTACCGACACGCTGAACCGTCAGGGGCTACGCGTGGTTTAAGACCCACTTTCACATTTAAG |
| KHU603 | CCAATAATGACATCGCCCGCATCCAGCCCAACCTCATGGCCTAAGCACTTGTCTCCTGTTTAC |
| KHU606 | TGCAAAGGCGCGTTACAGGA |
| KHU607 | GACGATGCGCTCTTTATGCA |
| 4308 | ACCGCGGTAAATGCGACTAT |
| 4309 | TGCCGCGACTTTCAGACA |
| 6970 | CCAGCAGCCGCGGTAAT |
| 6971 | TTTACGCCCAGTAATTCCGATT |
| 7763 | TCAGAAAATGATAAGCAGCATAAAAAA |
| 7764 | CCCTGACGATGGCTGTTCA |

**Supplementary references**

Choi, E., Choi, S., Nam, D., Park, S., Han, Y., Lee, J.S., and Lee, E.J. (2017) Elongation factor P restricts *Salmonella*'s growth by controlling translation of a Mg^2+^ transporter gene during infection. *Scientific reports* **7**: 42098.

Choi, E., Lee, K.Y., and Shin, D. (2012) The MgtR regulatory peptide negatively controls expression of the MgtA Mg^2+^ transporter in *Salmonella enterica* serovar Typhimurium. *Biochem Biophys Res Commun* **417**: 318-323.

Cromie, M.J., Shi, Y., Latifi, T., and Groisman, E.A. (2006) An RNA sensor for intracellular Mg(2+). *Cell* **125**: 71-84.

Datsenko, K.A., and Wanner, B.L. (2000) One-step inactivation of chromosomal genes in *Escherichia coli* K-12 using PCR products. *Proc Natl Acad Sci U S A* **97**: 6640-6645.

Fields, P.I., Groisman, E.A., and Heffron, F. (1989) A *Salmonella* locus that controls resistance to microbicidal proteins from phagocytic cells. *Science* **243**: 1059-1062.

Fields, P.I., Swanson, R.V., Haidaris, C.G., and Heffron, F. (1986) Mutants of *Salmonella* *typhimurium* that cannot survive within the macrophage are avirulent. *Proc Natl Acad Sci U S A* **83**: 5189-5193.

Lee, E.J., Pontes, M.H., and Groisman, E.A. (2013) A Bacterial Virulence Protein Promotes Pathogenicity by Inhibiting the Bacterium's Own F1Fo ATP Synthase. *Cell* **154**: 146-156.

Nam, D., Choi, E., Shin, D., and Lee, E.J. (2016) tRNA^Pro^-mediated downregulation of elongation factor P is required for *mgtCBR* expression during *Salmonella* infection. *Mol Microbiol* **102**: 221-232.

Park, S.Y., Cromie, M.J., Lee, E.J., and Groisman, E.A. (2010) A bacterial mRNA leader that employs different mechanisms to sense disparate intracellular signals. *Cell* **142**: 737-748.
